# Pollen derived macromolecules serve as a new class of ice-nucleating cryoprotectants

**DOI:** 10.1038/s41598-022-15545-4

**Published:** 2022-07-19

**Authors:** Kathryn A. Murray, Nina L. H. Kinney, Christopher A. Griffiths, Muhammad Hasan, Matthew I. Gibson, Thomas F. Whale

**Affiliations:** 1 Department of Chemistry, University of Warwick, Gibbet Hill Road, Coventry, CV4 7AL UK; 2 Department of Aquatic Resources, Institute of Marine Research, Swedish University of Agricultural Sciences, Turistgatan 5, 453 30 Lysekil, Sweden; 3 Ecology and Evolutionary Biology, School of Biosciences, University of Sheffield, Sheffield, S10 2TN UK; 4 Division of Biomedical Sciences, Warwick Medical School, University of Warwick, Gibbet Hill Road, Coventry, CV47AL UK

**Keywords:** Bioinspired materials, High-throughput screening, Atmospheric chemistry, Regenerative medicine

## Abstract

Cryopreservation of biological material is vital for existing and emerging biomedical and biotechnological research and related applications, but there remain significant challenges. Cryopreservation of cells in sub-milliliter volumes is difficult because they tend to deeply supercool, favoring lethal intracellular ice formation. Some tree pollens are known to produce polysaccharides capable of nucleating ice at warm sub-zero temperatures. Here we demonstrated that aqueous extractions from European hornbeam pollen (pollen washing water, PWW) increased ice nucleation temperatures in 96-well plates from ≈ − 13 °C to ≈ − 7 °C. Application of PWW to the cryopreservation of immortalized T-cells in 96-well plates resulted in an increase of post-thaw metabolic activity from 63.9% (95% CI [58.5 to 69.2%]) to 97.4% (95% CI [86.5 to 108.2%]) of unfrozen control. When applied to cryopreservation of immortalized lung carcinoma monolayers, PWW dramatically increased post-thaw metabolic activity, from 1.6% (95% CI [− 6.6 to 9.79%]) to 55.0% (95% CI [41.6 to 68.4%]). In contrast to other ice nucleating agents, PWW is soluble, sterile and has low cytotoxicity meaning it can be readily incorporated into existing cryopreservation procedures. As such, it can be regarded as a unique class of cryoprotectant which acts by inducing ice nucleation at warm temperatures.

## Introduction

Cryopreservation is an essential process in clinical, industrial and research settings, enabling long term storage of biological material. However, there are numerous instances where cryopreservation outcomes are inadequate for practical applications. For instance, cryopreserving cells of any type in the industry-standard 96-well plate format leads to significantly reduced post-thaw yields compared to vial-based freezing. Plates are routinely employed for high-throughput screening, as well as basic biomedical research, meaning methods for improving cryopreservation outcomes in them would be of great utility^1,2^.

The goal of any cryopreservation procedure is to cool biological material to a temperature low enough to inhibit chemical and biological processes. There are, broadly speaking, two methods employed for cryopreservation; ’slow’ freezing^3^ and vitrification^4^. Vitrification employs very fast cooling rates and high cryoprotectant concentrations to minimise the rate of ice crystal formation^4^. The present study is concerned only with slow freezing, which is the most commonly used approach for the storage of biological material. Slow freezing involves cooling at a known, steady rate in the presence of at least one cryoprotectant. The success of this approach relies on cooling fast enough to minimize the amount of time for which cells are exposed to potentially cytotoxic cryoprotectants and increased solute concentrations, but slow enough to allow cells to dehydrate, reducing the probability of fatal intracellular ice formation (IIF)^5,6^. The balance of these two factors gives optimal outcomes at some intermediate cooling rate, typically around 1 °C/min for mammalian cells^7^ although this is dependent on cell type and cryoprotectant, among other factors^6^.

Many different water-soluble substances have been found to be effective cryoprotectants, starting with the discovery, by Christopher Polge and coworkers, that inclusion of 30% glycerol in cell media substantially improves the condition of frozen sperm cells post-thaw^8,9^. The mechanisms of action of cryoprotectant molecules remain poorly understood for the most part, although many help reduce the probability of intracellular ice formation by altering the physical properties of aqueous solutions, for instance by increasing viscosity or slowing the kinetics of ice growth^10^. Some cryoprotectants may also help to protect cell membranes and organelles during freezing and thawing^10^.

An often-neglected factor for successful slow freezing is the desirability of inducing extracellular ice nucleation at relatively warm sub-zero temperatures. Ice nucleation at warm temperatures helps cells survive cryopreservation by increasing the opportunity for cell dehydration by transfer of water from the thermodynamically unstable supercooled liquid contents of cells to the thermodynamically stable ice outside cells. This process reduces the likelihood of damaging intracellular ice formation^11^. For small volumes, such as 96-well plate wells, deliberate nucleation control is critical, as smaller volumes of water have a much greater propensity to supercool than larger volumes^2,12^. If nucleation is left uncontrolled and occurs at cold temperatures, cells have less opportunity to dehydrate. The optimal temperature for ice nucleation remains unclear, and it is likely dependent on a variety of factors including cell type, vessel type and cryoprotectant choice, amongst others. Generally, it seems that nucleation at temperatures close to the melting point of the cryoprotectant solution will be better as this will reduce the likelihood of intracellular ice formation^11^.

Several methods for inducing ice nucleation in cryopreservation procedures have been developed^11^. For instance, crystals of ice can be introduced into cryopreservation solutions at warm supercooled temperatures, as was done in early studies on mammalian tissue culture^3,13^. Electrofreezing, where strong electric fields induce ice formation via a poorly understood mechanism, has been used^14,15^ as has laser induced nucleation^16^. So called shock freezing, where complex temperature ramps are used to induce ice nucleation, most likely by generating a strong temperature gradient in the cryopreservation volume allowing nucleation to occur without excessively supercooling most of the sample, has been used^17^. This method has been incorporated into commercial controlled-rate freezers^18^. Ultrasound can also induce ice nucleation and has been employed for freeze drying of proteins^19^. All of these methods require direct intervention with the material being cryopreserved and are therefore not intrinsically suited to scalable freezing of biological samples, particularly if acceptably effective protocols are already in use in laboratory or commercial contexts^11^. A small number of chemical nucleators, which are capable of nucleating ice near the melting point via heterogeneous nucleation, have also been deployed. These include Snomax™^20,21^, which is derived from the bacterial plant pathogen *Pseudomonas syringae*, cholesterol crystals^22^, encapsulated silver iodide^23^, biologically inert minerals^24^ and sand^25^. However, these existing chemical nucleators are usually insoluble and difficult to sterilise, or else are difficult to introduce into cryopreservation volumes in a reproducible fashion^11^. At present, there is no soluble, sterilisable nucleator suitable for cryopreservation which can be used without troublesome alteration to existing protocols.

Nature has often served as a source of inspiration for solutions to biological problems, particularly in the field of cryobiology. It is known that windborne pollen grains are able to nucleate ice, a property that has been investigated by atmospheric scientists^26–35^. Studies on the nucleation ability of silver birch (*Betula pendula*) pollen, which has been investigated in more detail than that of any other pollen, suggest that the entity responsible for nucleation separates easily from pollen grains when exposed to water, in contrast to the ice nucleating proteins associated with bacteria^28,29^. Once filtered to remove the pollen grains, the resultant solution is known as pollen washing water (PWW). It is thought that the ice nucleating component of PWW is a polysaccharide^35^. While PWW is known to nucleate ice well the biological function of the ice nucleating polysaccharide remains unclear but may be associated with either pollen cold-tolerance or the triggering of precipitation which might conceivably aid pollen dispersal^35^.

In this study we demonstrate that incorporating PWW into freezing solutions in 96-well plates substantially increases nucleation temperatures. Further, we report that addition of PWW from European hornbeam (*Carpinus betulus*) into conventional cryoprotectant mixtures significantly improves cryopreservation outcomes for Jurkat and A549 mammalian cell lines in 96-well plates and attribute the improved cryopreservation outcomes to the warmer nucleation temperatures. Since PWW is soluble, non-cytotoxic and easily sterilisable, it can be easily incorporated into existing cryopreservation methods with the aim of improving outcomes where nucleation temperature is presently a limiting factor. PWW can be regarded as a new class of cryoprotectant, with clear applications in biomedical research and industrial processes.

## Results and discussion

### The freezing temperature of pollen washing waters

**Figure 1:**
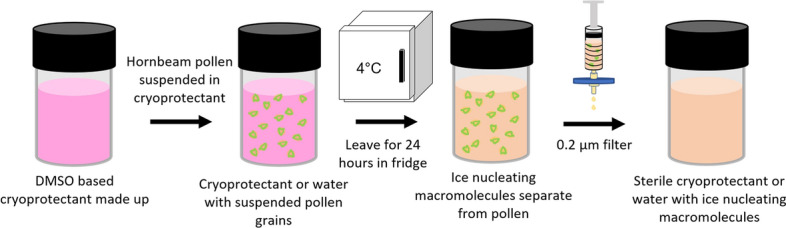
Schematic of preparation method for PWW.

A microliter droplet freezing assay, similar to that described by Whale et al.^36^, was used to investigate the ice nucleation ability of PWW made from the pollen of two tree species, silver birch (*Betula pendula*) and European hornbeam (*Carpinus betulus*). This technique cools an array of approximately 40 one microliter droplets at a rate of 2 °C/min. A camera is used to detect freezing of droplets during cooling, allowing the fraction of droplets frozen as a function of temperature to be determined. As shown in Fig. 1, PWW was prepared by suspending 2 wt%, of pollen grains in either complete cell culture medium or pure MilliQ water, leaving the suspension at 4 °C for 24 h, then filtering off the pollen grains, resulting in a sterile solution of the soluble materials associated with the pollen grains. PWWs for the microliter droplet freezing assay were prepared in MilliQ water rather than cryoprotectant solution.

**Figure 2:**
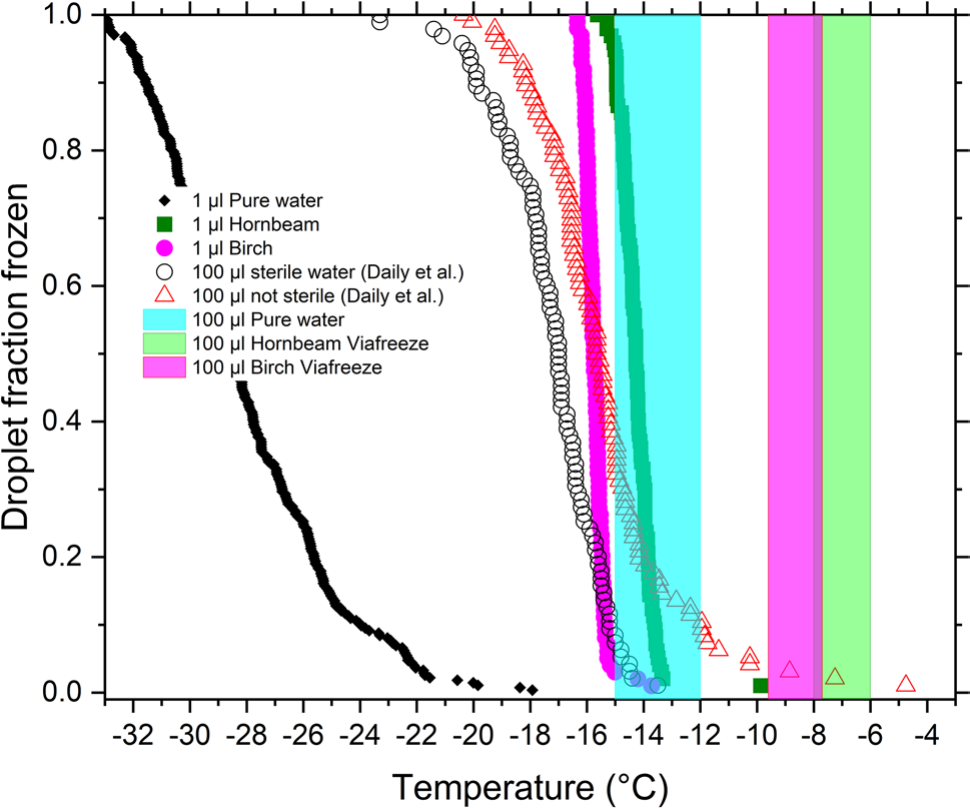
Plot of droplet fraction frozen as a function of temperature for pure water and PWWs in 1 µL and 100 µL, including measurements from Daily et al.^2^ for pure water. The shaded blocks represent estimated freezing ranges for 100 µL volumes of MilliQ water and PWWs from measurements made with embedded thermocouples.

Figure 2 shows droplet fraction frozen for 1 µL droplets of pure water and hornbeam and birch PWWs prepared in MilliQ water. Freezing occurred at median temperatures of − 14.2 °C in hornbeam PWW and − 15.1 °C in Birch PWW. In Fig. S1a the cumulative number of ice nucleators per pollen grain is compared to literature data^35^, indicating that the birch PWW used has a similar ice nucleation ability to previously tested samples. Our results for freezing of microliter droplets of PWW, and similar literature measurements, do not directly suggest that silver birch or hornbeam PWW is capable of nucleating ice consistently at temperatures above − 10 °C.

Gute and Abbatt^30^ froze 50 µL volumes of PWW and suspended pollen grains from various species in PCR plates. For silver birch pollen Gute and Abbatt^30^ used PWW solutions made with a mass concentration of pollen 20 times lower than we used here. In their ice nucleation experiment, which freezes droplets of 50 times greater volume than those in our microliter droplet freezing experiments, they found a median freezing temperature of − 14 °C, against − 15.1 °C in our experiments. This indicates that the two measurement approaches give broadly similar freezing temperatures for equivalent amounts of pollen. Gute and Abbatt^30^ found a median freezing temperature of − 6.5 °C for PWW from grey alder (*Alnus incana*) using equivalent conditions, and even warmer when higher pollen concentrations were used, showing that some pollens can induce ice nucleation at warm temperatures above − 10 °C.

It is commonly observed that larger quantities of nucleating materials nucleate ice at warmer temperatures than smaller quantities. As such, we measured the freezing temperature of 100 µL volumes of birch and hornbeam PWW made up using 2 wt% of pollen in standard 96-well plates. Freezing was detected using embedded thermocouples. Figure 2 shows the range of temperatures at which release of latent heat associated with freezing was detected in these experiments. In birch PWW freezing occurred between − 7.7 and − 9.6 °C and in hornbeam PWW between − 6.0 and − 8.0 °C. Figure 2 also shows that the freezing temperature for pure water in 96-well plates as determined using the same technique was between − 12 and − 15 °C. Literature measurements using an IR camera based technique determined slightly cooler freezing temperatures in 96-well plates^2,37^, also shown in Fig. 2. This difference is likely due to the intrinsic variability of uncontrolled freezing in 96-well plates^2^. 100 µL volumes of PWW freeze much closer to the melting point than 1 µL volumes of the same PWWs. We can conclude that PWW can raise the freezing temperature of water in 96-well plates to a point that might indicate potential utility for cryopreservation procedures.

### Why does PWW nucleate ice at warm temperatures?

The chemical nature of the ice nucleator in PWW remains somewhat unclear. Dreischmeier et al.^35^ demonstrated that the ice nucleating component of PWW is very likely a carboxylic acid-bearing polysaccharide of mass greater than 100 kDa. It was hypothesized that the ice nucleating polysaccharide in PWW may be either clusters or adducts of smaller ice binding polysaccharides also present in PWW, or that the ice binding polysaccharides may be fragments of the ice nucleating polysaccharides^35^. However, the actual structure of the polysaccharides has not been determined.

The ability of PWW to nucleate ice at temperatures as warm as − 6 °C, shown here and in Gute and Abbatt^30^ for grey alder pollen, is perhaps surprising. A 50 µL droplet of hornbeam PWW contains the soluble substances from approximately 150,000 pollen grains, while 1 µL droplets contain the material from only around 3000 grains, meaning that the chemical species capable of nucleating ice at − 6 °C is much rarer than that which nucleates at − 14 °C in microlitre droplet freezing experiments. Classical nucleation theory allows calculation of the critical nucleus size required for a stable ice crystal to form at a given temperature. It has been suggested that biological ice nucleators are likely to roughly match the size of the ice critical nucleus they cause to form^29^. Considering this, to induce ice nucleation at − 6 °C a nucleating molecule of around 2000 kDa is required^29^. By size exclusion chromatography the birch nucleator (active at around − 16 °C) has been found to be between 335 and 860 kDa^28^ larger than required for nucleation at − 16 °C, which requires a size of around 100 kDa, but insufficient for nucleation at the temperatures observed here in 96-well plates. It may be that that larger nucleators are produced by the plants only rarely, or that occasional chance dimer or multimer formation is required to produce the more effective nucleators. We propose that these very rare larger constructs are the most likely cause of ice nucleation at the warmer temperatures we have observed. It seems possible that grey alder produces more of these larger molecules hence the high ice nucleation activity observed for relatively smaller quantities of that pollen^30^. Analogously, differently sized aggregates of the ice nucleating protein produced by the bacteria *Pseudomonas syringae* are known to nucleate ice at different characteristic temperatures^38,39^, with larger aggregates nucleating at warmer temperatures.

Further measurements will be required to establish more precisely the temperature of activity and concentration of the ice nucleating molecules in PWW. Despite this, we can confidently state that ice nucleates in 100 µL volumes of both birch and hornbeam PWW at temperatures warm enough to potentially benefit cryopreservation outcomes in 96-well plates. The hornbeam PWW is the more effective nucleator of the two species, therefore we tested whether the ice nucleating capability of hornbeam PWW could provide a benefit in mammalian cell cryopreservation studies.

### Characterization of birch and hornbeam pollen washing water

We analyzed some relevant properties of PWW to evaluate its suitability as a cryoprotectant. To ensure that the PWW used was sterile, we prepared both birch and hornbeam PWWs in MilliQ water (not cell culture medium which contains antibiotics and would likely impede microbial growth) then attempted to culture both types of PWW on agar jelly. After 72 h there was no sign of microbial growth, confirming sterility.

A freezing point osmometer was used to measure the osmolality of both types of PWW made up in MilliQ water, which was found to be 30 ± 5 mOsmol/kg in both cases. Samples of both birch and hornbeam PWW were freeze dried and the residue weighed, establishing that for both types of PWW, approximately 0.7 ± 0.01% of the total mass of the samples is soluble material derived from the pollen. It was estimated that 36.6 ± 1.1% of birch pollen mass and 32.8 ± 1.1% of the hornbeam pollen mass was soluble and that the mass concentration of the solutions was 6.6 ± 0.2 mg/mL and 7.0 ± 0.2 mg/mL respectively. By assuming that no dissociated ionic compounds are present in PWW, the mean molecular mass of the constituents of PWW can be estimated from the osmolarity of the solution and the mass concentration. We calculate that in both cases the mean molecular mass is 230 ± 40 Da. As mentioned, the birch nucleator has been found to be between 335 and 860 kDa and molecules active at temperatures above − 10 °C may be larger still. This demonstrates that much of the content of the PWW is not the ice nucleating polysaccharide but other soluble compounds. The nature of these compounds is not known although it seems reasonable to suppose that monosaccharides and small polysaccharides are present in significant quantities. The low concentration of soluble material in PWW means it is very unlikely that use of PWW has any osmotic impact on cryopreservation processes.

### Cytotoxicity of PWW on A549 and Jurkat cells

Given the heterogeneous composition and biogenic origin of PWW, we assessed its cytotoxicity by treating adherent monolayers of A549 (immortalised human lung carcinoma) cells and suspended Jurkat (immortalised human T lymphocyte) cells with serial dilutions of PWW for 24 h, followed by measurement of cell metabolic activity by resazurin assay. These were chosen as they represent two of the most commonly frozen cell formats (monolayer and suspension). The metabolic activity of Jurkat cells treated with 100% (v/v) PWW for 24 h was reduced to 90.0 ± 6.6% relative to untreated controls (Fig. S3a). For all other concentrations tested [50% (v/v) to 3.125% (v/v)], metabolic activity remained above 99.0%. The metabolic activity of A549 cells decreased to 85.4 ± 8.5% when treated with 25% (v/v) PWW, which decreased to 68.1 ± 7.3% and 60.0 ± 3.5% when higher concentrations [50 and 100% (v/v)] of PWW were tested (Fig. S3b). Staining with calcein-AM and ethidium homodimer-1 (Live/Dead assay) was also used to assess the effect of PWW on A549 cells. Results showed there was no difference between any test condition and untreated controls (Fig. S4), suggesting that cell death was not increased at higher PWW concentrations. During the cryopreservation procedures the cells are only exposed to liquid PWW for around 10 min, which is insufficient for any detectable cytotoxic effect.

### Cryopreservation of Jurkat and A549 cells

**Figure 3:**
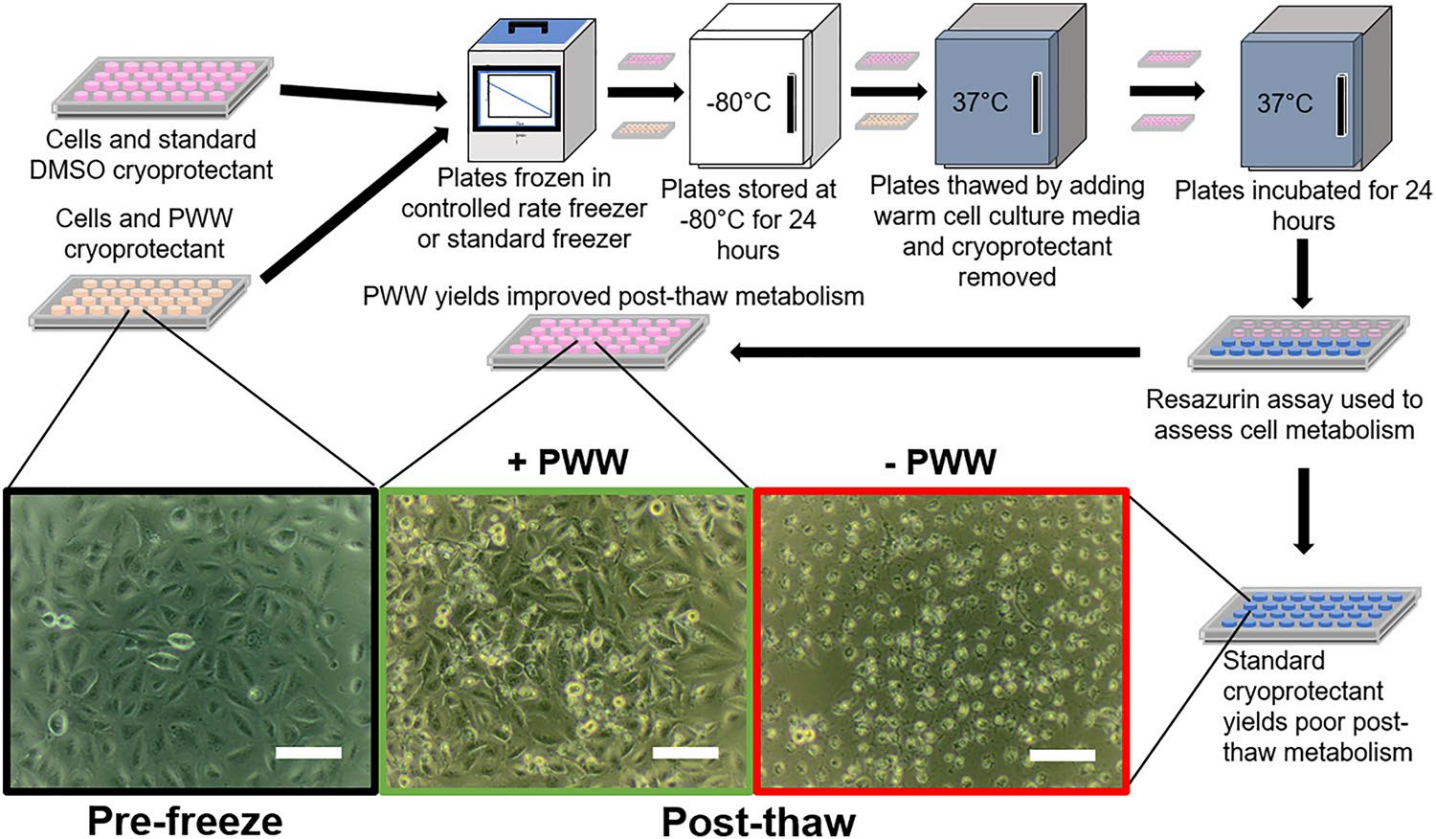
Schematic of cryopreservation procedure used to measure the effectiveness of PWW as a cryoprotectant. The micrographs show A549 cell monolayers before cryopreservation and after cryopreservation, with and without PWW. Scale bars indicate 100 µm.

As mentioned above, two different cell types, adherent A549 cell monolayers and suspended Jurkat cells, were selected for cryopreservation experiments. Stable cultures were produced by incubation of cells in appropriate cell media in 96-well plates. Figure 3 is a schematic of the cryopreservation procedure employed. Briefly, both cell types were exposed to 50 µL cryoprotectant solution containing 10% (v/v) DMSO for A549 cells and 5% (v/v) DMSO for Jurkat cells. This was made up in either complete cell medium control or in hornbeam PWW produced from complete cell medium as shown in Fig. 1. For each batch of cells, plates were divided into quadrants, two with the standard DMSO cryoprotectant and two with PWW cryoprotectant. A controlled rate freezer was used to cool plates from ambient temperatures to − 80 °C at nominal controlled rates of 0.5 °C/min and 2 °C/min. Plates were also placed directly onto the base of a − 80 °C freezer, a process hereafter referred to as ‘uncontrolled’ freezing, which replicates a method used in a typical laboratory without access to controlled rate freezers. The initial cooling rate this approach generates is − 9 °C/min, as discussed later. After storage for 24 h at − 80 °C, plates were thawed by addition of 100 µL/well of warm (37 °C) cell medium and placed in a 37 °C incubator. After 10 min the cryopreservation and thawing medium was removed and replaced with cell medium and the plates incubated for a further 24 h at 37 °C.

**Figure 4:**
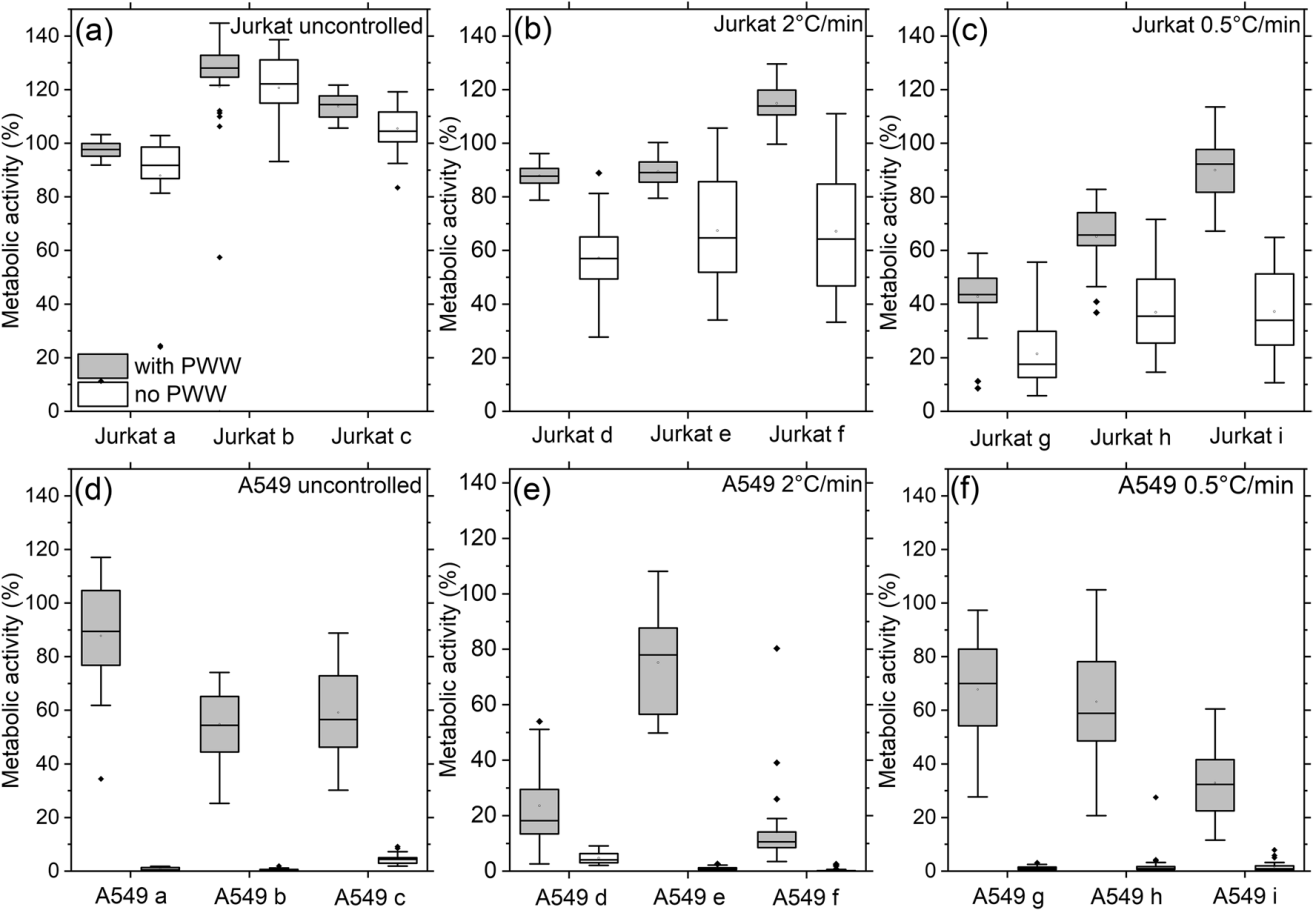
Cryopreservation enhancement in 96-well plates. (**a**–**f**) Boxplots of metabolic activity as measured by resazurin assay 24 h post-thaw for individual batches of A549 and Jurkat cells. The whiskers show the maximum and minimum values recorded for each dataset, the box shows the first and third quartiles, the centre line represents the median value found and dots show outliers. The number of wells measured for each experiment can be found in Table S1.

**Figure 5:**
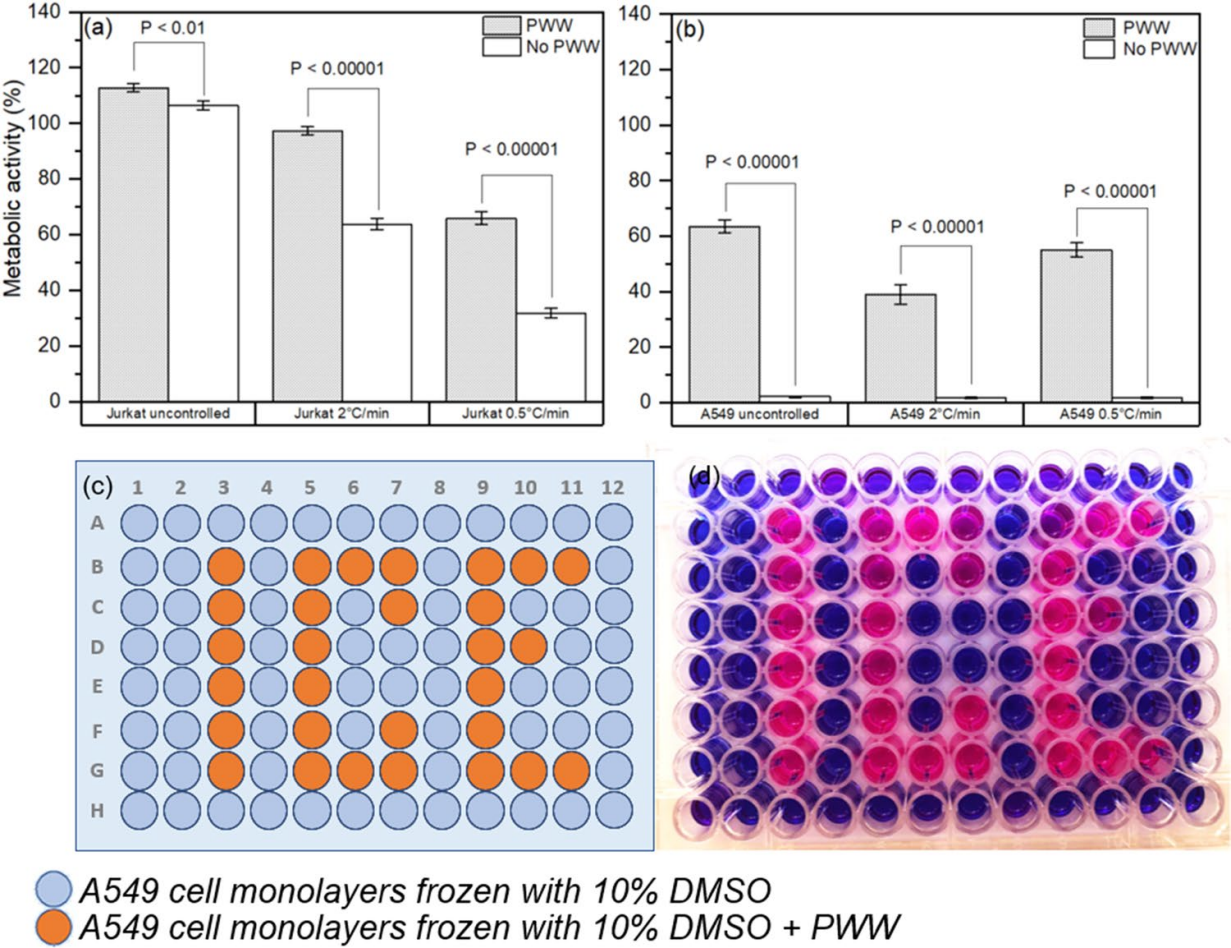
Overall cryopreservation enhancement in 96-well plates. (**a**) Mean post-thaw metabolic activity for Jurkat cells. (**b**) Mean post-thaw metabolic activity for A549 cells. Error bars are the standard error of the mean while the p-values are from 2-sample *t* tests comparing the conditions with PWW and without PWW for each cell type and cooling rate. (**c**) Plate map of A549 cell monolayers in a 96-well plate treated with either 10% DMSO (grey) or 10% DMSO in 50% PWW (orange). (**d**) Photograph of the 96-well plate following cryopreservation at an uncontrolled rate in a − 80 °C freezer for 24 h followed by thawing and incubation at 37 °C for a further 24 h and then treatment with resazurin solution. Resazurin solution (blue) is reduced to resorufin (pink) in the presence of metabolically active cells.

Addition of hornbeam PWW prior to freezing was found to significantly increase post-thaw metabolic activity, as measured by the resazurin assay, for both A549 (F = 809.0, p < 0.0001) and Jurkat (F = 81.51, p < 0.0001) cells. This result was found to be consistent across all three cooling rates. Data for each batch of cells are shown in Fig. 4a–f and pooled data are shown in Fig. 5a,b. Table S1 reports the number of wells frozen in each replicate along with post-thaw metabolic activities. The resazurin assay was chosen to allow a sufficiently large dataset to be produced in a reasonable amount of time, however, qualitatively similar results were observed in preliminary studies using trypan blue staining.

Some variation was observed between experimental repeats of metabolic activity measurements (Fig. 4a–f) therefore a linear mixed effect modelling framework was employed to appropriately account for this variation as a random effect. Model selection via Akaike Information Criterion (AIC) revealed that the most parsimonious model for both cell types accounted for both the presence and absence of PWW and the three freezing rates (Tables S2 and S3), demonstrating that both factors influenced metabolic activity. Specifically, post-thaw metabolic activity was found to be highest in the presence of PWW using the uncontrolled cooling rate for both cell types (Table 1). For A549 cells, use of PWW facilitates successful cryopreservation as post-thaw metabolic activity was close to zero when PWW was absent (Fig. 4d–f). For Jurkat cells, PWW increased post-thaw metabolic activity relative to no PWW in all three cooling rates, although the effect was less pronounced at the highest cooling rate. These results demonstrate that the addition of hornbeam PWW provides a significant increase in post-thaw viability for both cell types following cryopreservation in a convenient 96-well plate format.

**Table 1:** Fixed effect estimates and confidence intervals (lower = 2.5%–higher = 97.5%) for post-thaw A549 and Jurkat cell metabolic activity, extracted from the most parsimonious model (see Tables S2 and S3). All values are rounded to 2 decimal places.

	Cooling rate (°C/min)		
	0.5	2.0	Uncontrolled
**A549**			
No PWW	1.58 (− 6.63 to 9.79)	1.34 (− 6.92 to 9.58)	1.16 (− 7.20 to 9.46)
PWW	55.04 (41.64 to 68.44)	38.64 (25.00 to 52.25)	62.83 (48.76 to 76.83)
**Jurkat**			
No PWW	31.86 (26.50 to 37.21)	63.88 (58.53 to 69.24)	104.65 (99.29 to 110.01)
PWW	65.95 (55.12 to 76.78)	97.35 (86.52 to 108.18)	109.83 (99.00 to 120.66)

Thermocouples were embedded into cell freezing experiments to confirm that ice nucleation occurred at warmer temperature in wells containing PWW than those without, and to directly assess the cooling rate experienced by cells placed directly into the − 80 °C freezer. Fig. S2a shows temperature traces for individual thermocouples embedded in wells of the individual repeats using A549 cells for each of the three cooling conditions. In all cases one thermocouple was embedded in a location surrounded by wells containing PWW and the other thermocouple by wells containing normal cryoprotectant. The observed initial cooling rates were around 0.4 °C/min and 1.6 °C/min for the 0.5 °C/min and 2 °C/min nominal cooling rates respectively (Fig. S2b). For the uncontrolled cooling method, observed initial cooling rate was around 9 °C/min, although the cooling rate of the liquid contents of the plate is likely to be somewhat lower. It was clear that nucleation and subsequent freezing occurred at warmer temperatures in the presence of PWW across all cooling rates. These results are discussed further in the SI.

To demonstrate the efficacy of PWW as a cryoprotectant, A549 cell monolayers were treated in a specific pattern with either standard cryoprotectant containing 10% DMSO or PWW + 10% DMSO (Fig. 5c). The cells were then cryopreserved at an uncontrolled rate in a − 80 °C freezer for 24 h. Following thawing and incubation at 37 °C for 24 h, the medium was removed and replaced with resazurin solution to assess cell metabolic activity. Resazurin solution remained on the cells for 24 h to allow maximum visual difference between conditions to develop. Visual inspection demonstrated that only wells treated with PWW cryoprotectant + 10% DMSO were metabolically active post-thaw, reducing the resazurin (blue) to resorufin (pink; Fig. 5d). This demonstration shows that use of PWW allows control of cell health post-thaw and that use of a specific pattern does not impact the efficacy of PWW as a cryoprotectant.

We have shown that PWW is capable of consistently nucleating ice at temperatures above − 10 °C in 50–100 µL volumes, and that ice nucleation at these warm temperatures led to improved cryopreservation outcomes in the systems studied. Jurkat cells benefited from faster cooling rates, with a cooling rate of 9 °C/min (produced by placing plates directly in the freezer) generating the best outcomes, with or without nucleation control. The impact of varied cooling and thawing rates on Human Peripheral Blood T cell viability was investigated recently by Baboo et al.^40^ Jurkat cells are immortalized T cells so comparison with their results seems appropriate. Baboo et al.^40^ reported relatively small differences in cell viability and proliferation post-thaw, although it was found that if higher (10 °C/min) cooling rates were used, then slower (< 6.2 °C/min) warming rates were detrimental. Our method for warming is likely to generate a rate intermediate between the ‘slow thaw’ (6.2 °C/min) and the ‘standard thaw’ (45 °C/min) used by Baboo et al.^40^ so we might have expected better outcomes with our slower cooling rates, rather than the clear benefit of faster cooling rates we did observe. There are likely to be differences between primary T-cells and Jurkats that may be relevant. According to the two-factor hypothesis, faster cooling rates are favored when cells are able to dehydrate relatively quickly while still avoiding intracellular ice formation^41^, so it may be the Jurkat cells have higher membrane permeability or surface-area-to-volume ratio than primary T-cells.

The different sensitivities of the post-thaw metabolic activities of A549 and Jurkat cells to PWW are of interest. A549 cells benefit more from use of PWW than Jurkat cells. The most relevant difference between the cell types is that A549 cells are frozen as adherent monolayers while the Jurkat cells are frozen as suspended cells. It has been observed previously that some cell monolayers tend to detach from substrates during cryopreservation to the detriment of post-thaw cell health^42,43^. Detachment has been attributed to differential thermal expansion of the substrate and cell monolayer^44,45^. Daily et al.^2^ observed qualitatively that control of ice nucleation by seeding with ice helped reduce monolayer detachment in bovine granulosa cells cryopreserved in 96-well plates. Increasing the ice nucleation temperature may result in smaller temperature fluctuations during cooling as less latent heat is released when ice nucleates at warmer temperatures, which may reduce the impact of differential thermal expansion on monolayer detachment. Also, in experiments performed here, it is likely that ice forms above the cell monolayer because this is where the vast majority of PWW nucleators will be present. The same will be true of the experiments in Daily et al.^2^ where ice is introduced at the top of the well. In the absence of nucleation control, ice may be more likely to form on or near the monolayer itself, increasing the likelihood of displacement of the monolayer and damaging post-thaw outcomes. 

An alternative explanation is that propagation of ice between cells in monolayers may make avoidance of intracellular ice formation more critical in cell monolayers than in suspended cells. Acker et al.^46^ demonstrated that intracellular ice formation was much more prevalent in cells either attached to glass plates, in colonies on glass plates or in suspended spheroids than in suspensions of isolated cells. The implication of this is that some cells may tend to suffer intracellular ice formation at warmer temperatures than others and propagation of ice in grouped cells magnifies the negative impact of this. Control of ice nucleation by use of PWW will reduce the likelihood of IIF by favoring water loss from cell interiors earlier in the cooling process. Due to cell-to-cell ice propagation this may be much more important for cryopreservation of cell monolayers than suspended cells.

## Conclusions

To summarize, use of PWW during cryopreservation of suspended Jurkat and adherent A549 cells in 96-well plates gives significant improvements to cell metabolic activity post-thaw, which cannot be achieved using standard cryoprotectants (which have only a colligative impact on ice nucleation temperature). For adherent A549 cells, use of PWW effectively enables cryopreservation in 96-well plates as post-thaw metabolism is near-zero in the absence of PWW. For suspended Jurkat cells, use of PWW allows almost complete post-thaw cell recovery. Translation of such recovery to practical applications, especially for related immune cells which form the basis of emerging cell-based therapies such as chimeric antigen receptor T (CAR-T) cells, would be of great benefit. We have shown that PWW is capable of nucleating ice at temperatures above − 10 °C in 96-well plates and attribute the improved cellular outcomes to the increase in nucleation temperatures, in accord with the substantial literature suggesting that limiting supercooling improves outcomes during slow-cooling cryopreservation procedures^11^. Given that PWW is also sterile, as we have demonstrated, it represents a potential new class of soluble cryoprotectant that can be straightforwardly added to existing cryoprotectant mixtures with reasonable expectation of improved outcomes, particularly when volumes of less than 1 mL are frozen. Future work should be directed at isolating, sequencing and, ideally, synthesizing the ice nucleating polysaccharide present in PWW, which would likely find wide ranging applications.

## Materials and methods

### Preparing pollen washing water (PWW)

To produce PWW for ice nucleation experiments, 0.2 g of pollen from either silver birch (*Betula pendula*) or European hornbeam (*Carpinus betulus*), both purchased from Pharmallerga, was suspended in 10 mL MilliQ water overnight, then filtered through a 0.2 µm filter. This procedure produces a transparent yellow liquid free from pollen grains. For cell cryopreservation experiments, PWW was prepared as above using complete cell culture medium in place of water.

### Measurement of ice nucleation temperatures

To determine the freezing temperatures of microlitre droplets, a droplet freezing assay based on that described by Whale et al.^36^ was used. Arrays of approximately 40 one microlitre droplets were pipetted onto silanised glass slides (Hampton Research HR3-231) and cooled using an aluminium Peltier-driven cold stage. The Peltier was controlled by a Meerstetter TEC-1091 reading a Netsushin 1 mm diameter PT-100 embedded directly under the glass slide. A recirculating chiller was used as backing cooling for the cold stage. Separately, a PicoTech PT-104 was used to monitor two more PT100s embedded in different locations under the glass slide. A camera monitors freezing of droplets allowing the fraction of droplets frozen at a given temperature to be determined.

To investigate nucleation temperatures directly in standard Society for Biomolecular Sciences flat-bottom 96-well plates (Corning), a Cytiva VIA Freeze™ Uno (VFU) controlled rate freezer was used to cool plates containing 92 100 µL aliquots of MilliQ water, birch PWW and hornbeam PWW at a nominal rate of 2 °C/min. T-type thermocouples were embedded in the remaining 4 wells using pressure sensitive putty. The wells containing thermocouples were central to the four quadrants of the plate. The thermocouples were calibrated against Netsushin PT-100s. A thermocouple logger integrated into the VFU was used to monitor the temperature of the plates during cooling. Freezing can be detected by deviation of the steady temperature ramp caused by the release of latent heat during water freezing as shown in Fig. S1b. The same method was used to monitor freezing temperatures and cooling rates during a subset of cryopreservation procedures also. It should be noted that the deviation from the initial steady cooling rate is associated with freezing, as opposed to nucleation and that there will be a degree of lag between the nucleation event and detection of resultant latent heat associated with the thermal conductivity of the plate and the putty used. It is anticipated that at cooling rates of 0.5 °C/min and 2 °C/min these effects are quite small but they may lead to an overestimate of the degree of supercooling reached before nucleation.

### Cell culture

Human Caucasian lung carcinoma cells (A549) (ECACC 86012804) and Jurkat E6.1 cells (ECACC 88042803) were obtained from the European Collection of Authenticated Cell Cultures and cultured in complete culture medium consisting of F-12K (Gibco) or Advanced RPMI 1640 (Gibco), respectively, containing 10% (v/v) fetal bovine serum (FBS) (Merck), 100 units mL^−1^ penicillin, 100 μg mL^−1^ streptomycin and 250 ng mL^−1^ amphotericin B (Gibco). Cells were maintained at 37 °C, 5% CO_2_ and sub-cultured every 3–4 days.

### Cryopreservation of A549 cells in 96-well plates

Adherent A549 monolayers were washed with sterile phosphate buffered saline (PBS) then dissociated using 0.25% trypsin plus 1 mM ethylenediaminetetraacetic acid (EDTA) in balanced salt solution. Following centrifugation at 180 g for 5 min, a sample of A549 cells was diluted 1:1 in 0.4% trypan blue and counted using a haemocytometer. Cells were resuspended at a cell density of 2 × 10^5^ cells/mL. 100 µL of cell suspension (2 × 10^4^ cells/well) was added to individual wells of a flat-bottom 96-well plate (Corning) and cells were allowed to adhere overnight. The following day, cryoprotectant solutions were prepared, consisting of 10% (v/v) DMSO in either 50% (v/v) PWW or complete cell medium. Medium was removed from wells and 50 µL cryoprotectant was added to each well. Plates were either placed directly in a freezer set to − 80 °C for 24 h or placed in a VIA Freeze™ Uno controlled rate freezer and frozen at either 0.5 or 2 °C/min until the plate reached − 80 °C. At this point plates were transferred to a freezer set to − 80 °C for 24 h.

To thaw, 100 µL of warm (37 °C) complete cell medium was added to each well and the plate was incubated at 37 °C, 5% CO_2_ for 10 min. The medium was replaced with 100 µL complete medium and non-frozen control cells were added to the plate at 1 × 10^5^ cells/mL in 100 µL. The plate was then incubated for 24 h at 37 °C, 5% CO_2_. After 24 h the supernatant was removed and 100 µL resazurin solution (prepared in phenol red-free F-12 medium containing 10% (v/v) FBS, 100 units mL^−1^ penicillin, 100 μg mL^−1^ streptomycin and 250 ng mL^−1^ amphotericin B) was added to each well. The plate was incubated at 37 °C, 5% CO_2_ and the absorbance was monitored until 70% reduction was achieved for non-frozen controls.

### Cryopreservation of Jurkat cells in 96-well plates

A sample of Jurkat cells was diluted 1:1 in trypan blue and counted using a haemocytometer. Following counting, cells were centrifuged at 180×*g* for 5 min and resuspended at a cell density of 2 × 10^6^ cells/mL. 25 µL of cell suspension was added to individual wells of a U-bottom 96-well plate (5 × 10^4^ cells/well). DMSO [10% (v/v)] was added to solutions of either PWW or complete cell medium to prepare cryoprotectant solutions at 2 × final concentration. 25 µL of 2 × cryoprotectant solution was added to each well and mixed, final PWW concentration 50% (v/v) and 5% DMSO (v/v). Plates were either placed directly in a freezer set to − 80 °C for 24 h or placed in a VIA Freeze™ Uno controlled rate freezer and frozen at either 0.5 or 2 °C/min until the plate reached − 80 °C. At this point plates were transferred to a freezer set to − 80 °C for 24 h.

To thaw, 100 µL of warm (37 °C) complete cell medium was added to each well and the plate was incubated at 37 °C, 5% CO_2_ for 10 min, then centrifuged (730×*g* for 5 min) to pellet cells. The supernatant was removed and cells were resuspended in 100 µL fresh medium followed by 24 h incubation at 37 °C, 5% CO_2_. After 24 h, non-frozen controls were prepared at 5 × 10^5^ cells/well and added to the plate in 100 µL. The plate was centrifuged (730×*g* for 5 min) to pellet cells then the medium was replaced with 100 µL resazurin solution (prepared in phenol red-free RPMI 1640 medium containing 10% (v/v) fetal bovine serum (FBS), 100 units mL^−1^ penicillin, 100 μg mL^−1^ streptomycin and 250 ng mL^−1^ amphotericin B). The plate was incubated at 37 °C, 5% CO_2_ and the absorbance monitored until 70% reduction was achieved for non-frozen controls.

### Toxicity of pollen washings on A549 cells

A549 cells were added to a flat-bottom 96-well plate at a density of 1 × 10^5^ cells/mL (1 × 10^4^ cells/well) and allowed to adhere overnight. PWW were prepared in complete cell medium as previously described. Serial dilutions of PWW were prepared by diluting the stock solution 1:1 in complete cell culture medium. Solutions were added at 100 µL/well and the plate was incubated at 37 °C, 5% CO_2_ for 24 h. After 24 h the supernatant was removed and cell metabolic activity was assessed by the resazurin assay, where absorbance was monitored until 70% reduction was achieved relative to media-only controls.

### Toxicity of pollen washings on Jurkat cells

Jurkat cells were prepared at a cell density of 5 × 10^5^ cells/mL and 100 µL of cells was added to individual wells of a U-bottom 96-well plate (5 × 10^4^ cells/well). The plate was centrifuged at 730×*g* for 5 min and the supernatant was removed. PWW were prepared in complete cell medium as previously described. Serial dilutions of PWW were prepared by diluting the stock solution 1:1 in complete cell culture medium. Solutions were added at 100 µL/well and the plate was incubated at 37 °C, 5% CO_2_ for 24 h. After 24 h the plate was centrifuged at 730×*g*, the supernatant was removed and cell metabolic activity was assessed by the resazurin assay, where absorbance was monitored until 70% reduction was achieved relative to medium-only controls.

### Live/dead assay of A549 cells

A549 cells were added to a flat-bottom 96-well plate at a density of 1 × 10^5^ cells/mL (1 × 10^5^ cells/well) and allowed to adhere overnight. PWW solutions were prepared in complete cell medium as previously described. Serial dilutions of PWW were prepared by diluting the stock solution 1:1 in complete cell culture medium. PWW solutions were added at 100 µL/well and the plate was incubated at 37 °C, 5% CO_2_ for 24 h. After 24 h, control cells were treated with 70% methanol in deionised water for 15 min at 37 °C as a ‘dead’ control. Medium was removed from all wells and cells washed with 100 µL PBS. A live/dead solution was prepared by adding 2.5 µM calcein-AM and 15 µM ethidium homodimer-1 to 5 mL sterile PBS and vortexed to mix. 100 µL of the live/dead solution was added to each well and the plate was incubated at room temperature protected from light for 30 min. After 30 min, phase contrast and fluorescence images were captured. Images were captured on an Olympus CKX41 microscope with pE-300-W LED illumination and a XC30 camera. For fluorescence images a 535 nm excitation filter with a 50 nm bandwidth and a 645 nm emission filter with 75 nm bandwidth were used. Three wells were analysed for each condition. Image analysis was performed using ImageJ software, version 1.52.

### Statistical analysis

The relationship between normalised metabolic rate and the two explanatory variables (presence or absence of pollen and freezing rate) was analysed using linear mixed effect models. Linear mixed effect models were fitted by restricted maximum likelihood (REML) and were used to ensure that any variability associated with the three experimental repeats could be appropriately considered in the random effects structure of the model. Stepwise model selection was conducted using ANOVA and Akaike information criterion (AIC). Model fit was assessed via visual inspection of the residuals. Models were fit separately to each cell line (A549 and Jurkat). All models were fitted in R (R Core Team, 2020; version 4.0.2) using the lme4 package^47^. Data visualization was conducted in OriginPro 2021b.

## Supplementary Information

Supplementary Information.

## Data Availability

The datasets generated and analysed during the current study are available on the Warwick Research Archive Portal (WRAP) repository at http://wrap.warwick.ac.uk/165506/.
