# Supplementary material for: Pollen derived macromolecules serve as a new class of ice-nucleating cryoprotectants
Source: Sci Rep. 2022 Jul 19;12:12295. doi: 10.1038/s41598-022-15545-4 (PMC9296471; doi:10.1038/s41598-022-15545-4)
Supplement: Supplementary file 1 — Supplementary Information. [file 41598_2022_15545_MOESM1_ESM.pdf]

Supplementary information

Pollen derived macromolecules serve as a new class of ice-nucleating cryoprotectants

Kathryn A. Murray,[a] Nina L. H. Kinney,[a] Christopher A. Griffiths,[b,c] Muhammad Hasan,[a,d] Matthew I. Gibson,[a,d] Thomas F. Whale[a]*

[a]Department of Chemistry, University of Warwick, Gibbet Hill Road, Coventry, CV4 7AL, UK

[b]Swedish University of Agricultural Sciences, Department of Aquatic Resources, Institute of Marine Research, Turistgatan 5, SE-453 30, Lysekil, Sweden

[c]Department of Animal and Plant Sciences, University of Sheffield, Sheffield, S10 2TN, UK

[d]Warwick Medical School, University of Warwick, Gibbet Hill Road, Coventry, CV47AL, UK

Section S1: Freezing temperatures of water and PWW in microliter drops and 96-well plates

To directly compare our data with literature measurements made by Dreischmeier et al. [1] we have calculated the cumulative number of ice nucleators per pollen grain on cooling from 0°C to temperature T, called $n_n(T)$ using the equation[1]:

$$n_n(T) = \frac{-\ln(1 - f(T))}{n}$$

where f is the the droplet fraction frozen at temperature T and n is the number of pollen grains which contributed to the PWW in each droplet. This is a non-time dependent representation of ice nucleation, which does not consider the stochasticity of the nucleation process. To calculate n_n both pollens were assumed to have a density of 1 g/cm^3. Hornbeam pollen grains were taken to have a diameter of 33 µm [2] and birch pollen grains 24 µm [1]. These results, along with literature data from Dreischmeier et al.[1], are presented in Fig. S1 (a).

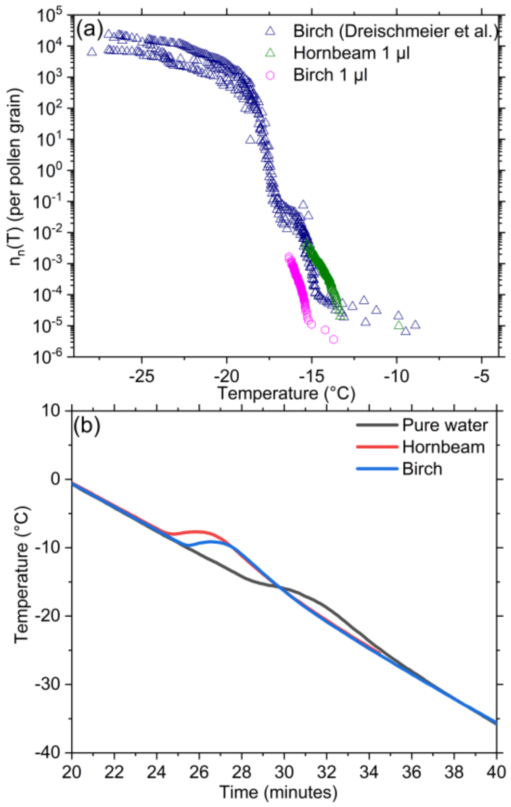

Fig S1 (a) Plot of cumulative number of ice nucleators per pollen grain for literature data from Dreischmeier et al.[1] and measurements made in this study on birch and hornbeam PWW. (b) Temperature trace from thermocouples embedded in 96-well plates containing 92 100 µL volumes of the two PWWs and MilliQ water.

Accurate measurement of freezing temperatures in the types 96-well plates used for cell biology is challenging, and to date has only been accomplished using complex infrared thermometry[3-5]. We instead used embedded thermocouples to measure plate temperature during cooling. Briefly, 92 wells of a 96-well plate were filled with 100 µL of MilliQ water, birch PWW or hornbeam PWW. The remaining four wells, one in the center of each quadrant of the 96-well plate, had thermocouples embedded in them using pressure sensitive putty. The plates were cooled at 2°C/min using a Cytiva ViaFreeze Uno (VFU) controlled rate freezer, with thermocouple temperature monitored by a

thermocouple reader built into the VFU. Freezing was detected by deviation from the set cooling rate as indicated in Fig. S1 (b). The range of freezing temperatures is also indicated by shaded areas on Fig. 2. It should be noted that this technique detects changes in temperature caused by the latent heat released by the ice crystallization which follows the nucleation event in each liquid volume, rather than the nucleation event itself.

It is likely that the wells containing thermocouples are consistently slightly cooler than the wells containing liquid as the heat capacity of the putty used to secure the thermocouple is lower than that of water. Once all wells in the vicinity of a measuring thermocouple are frozen, latent heat will stop being produced and the temperature of the thermocouple will start to return to the temperature set by the controlled rate freezer. As such, we have estimated the range of freezing temperatures as being the period during which rate of cooling is decreasing. In this way, we estimate that, in the 96-well plates used for this study, freezing occurs in MilliQ water between -12°C and -15°C, in birch PWW between -7.7°C and -9.6°C and in hornbeam PWW between -6.0°C and -8.0°C, which is a substantial increase in a cryobiological context. These are the ranges shown in Fig. 2 along with literature data for nucleation temperatures for MilliQ water frozen in 96-well plates, as determined by an IR camera-based technique[3,6].

These literature measurements detected nucleation events between about -13°C and -22°C, with most events between -15°C and -17°C, colder than our measurements. It is likely that the thermocouple technique we have used has limited sensitivity to isolated freezing events, compared to the IR camera technique or camera based techniques which individually detects freezing in each well. This means sporadic warmer and colder events may have occurred in our experiments but were not detected. As regards the observed difference in freezing temperature of pure water between our experiments and literature measurements, it is known that freezing temperatures in 96-well plates are highly variable [3]. It may be that the water and well plates we used in this study tend to nucleate ice at higher temperatures than those in the study we are comparing to due to differences in water purity or nucleating ability of the plates themselves. Nevertheless, both PWWs are clearly capable of consistently inducing freezing at temperatures above -10°C in 100 µL droplets, warm enough to expect a benefit for cryopreservation procedures.

Section S2. Measurement of cooling rate and nucleation temperature during cell cryopreservation in 96-well plates.

As mentioned in the main text T-type thermocouples read by a thermocouple reader incorporated into the Cytiva Viafreeze Uno (VFU) were embedded into wells of 96 well plates during A549 cryopreservation, in the same manner as described in the section above. This was done for the nominal 0.5°C/min and 2°C/min cooling rates and the uncontrolled cooling achieved by placing the plate onto the base of a -80°C freezer.

Temperatures traces for these measurements are shown in Fig. S2(a) The directly measured cooling rates for the 0.5°C/min and 2°C/min experiments are slightly lower than the cooling rate of the VFU plate. This can be seen most easily in Fig. S3(b), which shows the rate of change of temperature as a function of temperature for the data in Fig. S3(a). This lag is likely due to imperfect thermal contact between the 96-well plate and cooling plate.

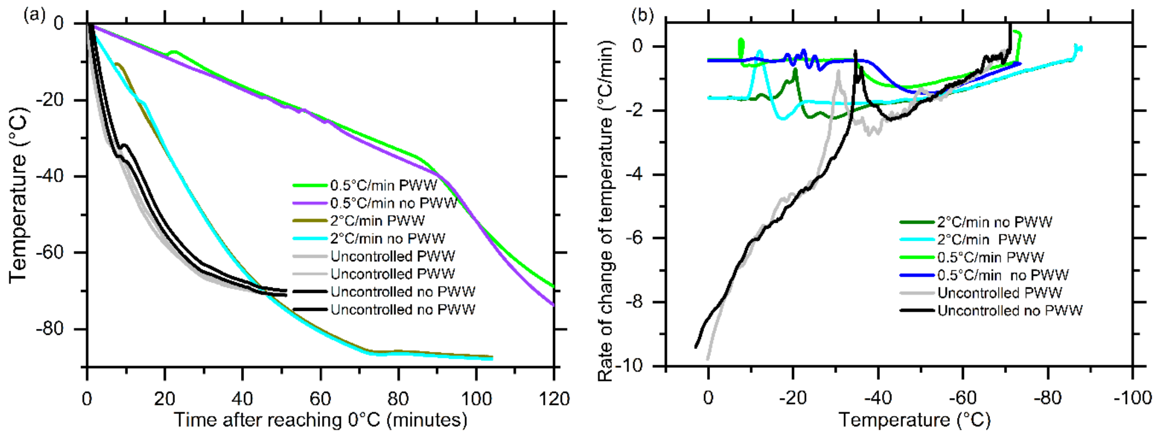

Fig. S2 (a) Traces of temperatures of individual wells of the samples A549 c, d and g during cryopreservation. (b) rate of change of temperature as a function of temperature for the data shown in (a).

Freezing temperatures in the 2°C/min experiment are consistent with the freezing temperatures observed in PWW made in pure water as freezing point osmometry of the cryoprotectant mixture used implies a melting point for the cryoprotectant of -4.2°C, similar to the temperature shift observed between the pure water and cryoprotectant cases. The release of latent heat due to freezing occurred around 1.5°C warmer in the 0.5°C/min experiment. This is likely due to two factors. Firstly, the thermocouples likely cool down faster than the well containing water, so there may be less lag in the 0.5°C/min experiment. Secondly, nucleation is stochastic in nature and the greater time allowed by a slower cooling rate will slightly favor nucleation at warmer temperatures. For both the controlled cooling rates the wells without PWW froze at cooler and more variable temperatures. Initial cooling rate as measured for the uncontrolled rate experiment was around -9°C/min. However, this varied substantially between wells, indicating that different parts of the plate experienced different cooling rates. Additionally, it is clear that the temperature of the solution is lagging substantially behind the temperature of the thermocouple in this experiment. It is very unlikely that the water reached -30°C before nucleation occurred in the PWW wells, as would appear be the case, because this is close to the homogenous nucleation temperature for water volumes of this size (10, 11). As such -9°C/min should be regarded as a maximum possible cooling rate. The latent heat release of freezing in the PWW wells occurred around the same amount of time after reaching 0°C as in the 2°C/min, suggesting that the initial cooling rate in the uncontrolled experiment may not in fact be a great deal faster 2°C/min, although the time taken to reach -70°C is likely substantially shorter than for the 2°C/min experiment. Irrespective, it is clear that in all cases PWW induces ice nucleation in 96-well plates at a warmer temperature than when PWW is absent as can be seen from the shifted changes from steady cooling rates in Fig. S2(b).

Section S3. Supplemental figures and tables

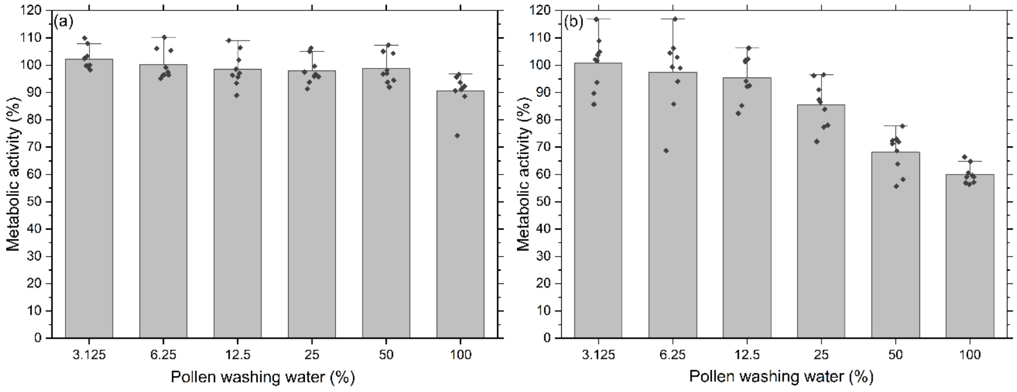

Figure S3. Cytotoxicity testing of Hornbeam PWW. (a) Jurkat cells and (b) A549 cells were treated with solutions of Hornbeam PWW from 3.125 to 100 % (v/v) for 24 hours at 37 ºC, 5 % CO_2. Metabolic activity was assessed by resazurin assay and compared to untreated controls. Data represents three biological replicates with three technical replicates.

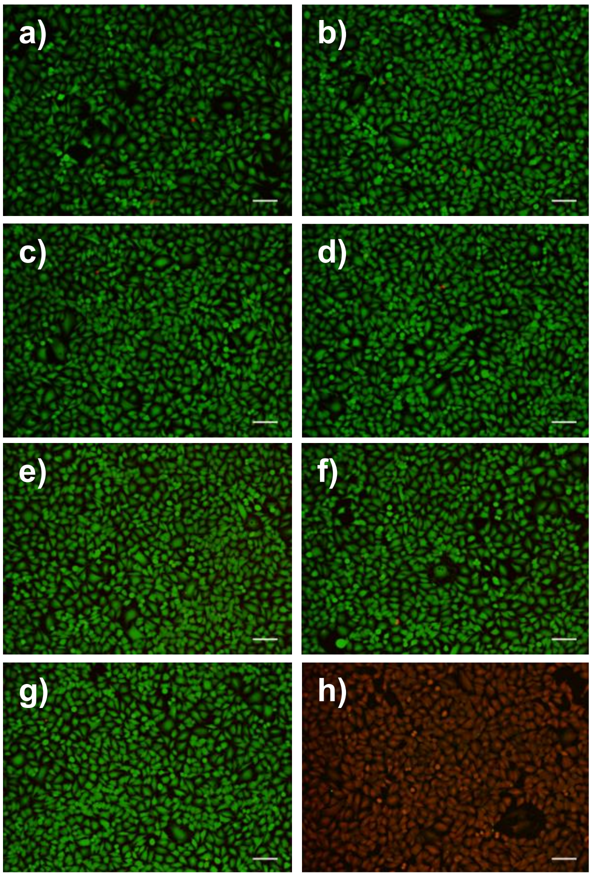

Fig. S4. Live/Dead assay of A549 cells treated with Hornbeam pollen washings. Cells were treated with solutions of PWW from 0 to 100 % (v/v) in complete cell media for 24 hours at 37 ºC, 5 % CO_2. a) 100 %, b) 50.0 %, c) 25.0 %, d) 12.5 %, e) 6.25 %, f) 3.13 %, g) live cell control, h) dead cell control by treatment with 70 % methanol in deionised water for 15 minutes at 37 ºC. Scale bar 100 µm.

Table S1. Conditions and post-thaw metabolic activity for cryopreservation experiments, with and without hornbeam PWW

**Cell type**	**Cooling rate**	**Repeat**	**Number of wells with PWW**[(a)]	**Number of wells without PWW**	**Post-thaw metabolic activity with PWW (% of unfrozen control)** [(b)]
Jurkat	Uncontrolled	1	28	30	127.8
Jurkat	Uncontrolled	2	30	30	113.7
Jurkat	Uncontrolled	3	30	28	94.7
Jurkat	0.5°C/min	1	30	30	42.7
Jurkat	0.5°C/min	2	30	30	65.1
Jurkat	0.5°C/min	3	30	30	90
Jurkat	2°C/min	1	30	30	87.8
Jurkat	2°C/min	2	30	30	89.4
Jurkat	2°C/min	3	30	30	114.8
A549	Uncontrolled	1	16	14	87.7
A549	Uncontrolled	2	30	30	54.8
A549	Uncontrolled	3	28	28	59.1
A549	0.5°C/min	1	28	28	33.0
A549	0.5°C/min	2	30	29	67.7
A549	0.5°C/min	3	30	30	63.1
A549	2°C/min	1	22	22	23.6
A549	2°C/min	2	30	30	75.2
A549	2°C/min	3	30	30	14.1

(a) Wells in a 8 x 12 96-well plate, (b) Metabolic activity measured by resazurin assay for 4 hours at 37 °C and normalised relative to unfrozen controls

Table S2. Model selection table via AIC for A549 cell line

Model formula	AIC	ΔAIC[(a)]	R^2 value[(b)]
nMR ~ (1|exp.no)	4746.2	-	0.029
nMR ~ Pollen + (1|exp.no)	4251.9	-494.3	0.653
nMR ~ Freezing rate + (1|exp.no)	4737.2	-9	0.052
nMR ~ Pollen + Freezing rate + (1|exp.no)	4220.9	-525.3	0.675
**nMR ~ Pollen * Freezing rate + (1|exp.no)**	**4187.1**	**-559.1**	**0.700**

[(a)]ΔAIC values are reported as differences from the most simplistic model. The most parsimonious model is shown in bold. R^2 squares are reported as the conditional (including both fixed and random effects), [(b)]R squared and are calculated using the *r.squared.GLMM* function in the 'MuMIn' package[7] in R.

Table S3. Model selection table via AIC for Jurkat cell line.

Model formula	AIC	ΔAIC[(a)]	R^2 value[(b)]
nMR ~ (1|exp.no)	5328.3	-	0.005
nMR ~ Pollen + (1|exp.no)	5254.0	-74.3	0.137
nMR ~ Freezing rate + (1|exp.no)	4946.3	-382.0	0.515
nMR ~ Pollen + Freezing rate + (1|exp.no)	4777.8	-550.5	0.646
**nMR ~ Pollen * Freezing rate + (1|exp.no)**	**4715.9**	**-612.4**	**0.686**

[(a)]ΔAIC values are reported as differences from the most simplistic model. The most parsimonious model is shown in bold. R^2 squares are reported as the conditional (including both fixed and random effects), [(b)]R squared and are calculated using the *r.squared.GLMM* function in the 'MuMIn' package[7] in R.

References

1 Dreischmeier, K., Budke, C., Wiehemeier, L., Kottke, T. & Koop, T. Boreal pollen contain ice-nucleating as well as ice-binding 'antifreeze' polysaccharides. *Scientific Reports* **7**, 41890, doi:10.1038/srep41890 (2017).

2 Halbritter, H., Schneider, H., Sam, S. & Auer, W. *Carpinus betulus. In: PalDat - A palynological database.*, https://www.paldat.org/pub/Carpinus_betulus/306095;jsessionid=DC05B66A833DA72103B86ED50A096307; accessed 2022-05-08

3 Harrison, A. D. *et al.* An instrument for quantifying heterogeneous ice nucleation in multiwell plates using infrared emissions to detect freezing. *Atmos. Meas. Tech.* **11**, 5629-5641, doi:10.5194/amt-11-5629-2018 (2018).

4 Zaragotas, D., Liolios, N. T. & Anastassopoulos, E. Supercooling, ice nucleation and crystal growth: a systematic study in plant samples. *Cryobiology* **72**, 239-243, doi:https://doi.org/10.1016/j.cryobiol.2016.03.012 (2016).

5 Kunert, A. T. *et al.* Twin-plate Ice Nucleation Assay (TINA) with infrared detection for high-throughput droplet freezing experiments with biological ice nuclei in laboratory and field samples. *Atmos. Meas. Tech.* **11**, 6327-6337, doi:10.5194/amt-11-6327-2018 (2018).

6 Daily, M. I. *et al.* Cryopreservation of primary cultures of mammalian somatic cells in 96-well plates benefits from control of ice nucleation. *Cryobiology* **93**, 62-69, doi:https://doi.org/10.1016/j.cryobiol.2020.02.008 (2020).

7 Bartoń, K. *MuMIn: Multi-Model Inference. R package version 1.43.17.*, <https://CRAN.R-project.org/package=MuMIn> (2020).
